# Jejunal Intussusception Due to an Adenocarcinoma

**DOI:** 10.7759/cureus.79219

**Published:** 2025-02-18

**Authors:** Carolina Silva, Adelaide Gomes da Costa, Ágata Ferreira

**Affiliations:** 1 General Surgery Department, Centro Hospitalar do Oeste, Caldas da Rainha, PRT

**Keywords:** adenocarcinoma, adult intussusception, iron deficiency anemia, jejuno-jejunal intussusception, small bowel

## Abstract

Intussusception occurs when a segment of the intestine telescopes into a neighboring part of the bowel. While it is uncommon in adults, unlike in children, when it does occur, a tumor is usually the underlying cause. Here, we report a rare case of a male patient diagnosed with jejunal adenocarcinoma, who initially presented with iron deficiency anemia. Intussusception was identified during surgery, and a laparoscopic resection of the affected jejunal segment was carried out.

## Introduction

Small bowel cancers (SBCs) are uncommon, accounting for only 1-3% of all gastrointestinal cancers [1]. These tumors often present with minimal and nonspecific symptoms, such as gastrointestinal bleeding, iron deficiency anemia, abdominal discomfort, nausea, and weight loss [1]. Rarely, they may present with complications like intestinal perforation or intussusception, which may need urgent surgical intervention [2]. Intussusception, which is a condition where a part of the intestine slides into an adjacent section, is a particularly difficult diagnosis to make and is often only detected during surgery. Given the increased risk of malignancy in adults, resection should be considered, emphasizing the importance of early diagnosis [3].

The duodenum is the most common location for SBCs (57%), followed by the jejunum (29%)[4] and ileum (13%) [2]. Early-stage SBC is most effectively treated through complete surgical resection [2].

We present the case of a 64-year-old patient diagnosed with jejunal adenocarcinoma, who initially presented with iron deficiency anemia. During surgery, a jejunal intussusception, caused by the tumor, was discovered.

## Case presentation

A 64-year-old patient with a history of hypertension and atrial fibrillation was referred for a gastroenterology evaluation due to iron deficiency anemia (Table 1). He was under rivaroxaban, but there were no visible signs of bleeding. The patient did not report any other symptoms.

**Table 1:** Laboratory results

Laboratory investigation	Patient’s results (reference range)
Hemoglobin	9.8 g/dL (13.6-18)
Hematocrit	29.7% (39.8-52)
Mean corpuscular volume	85.5 fL (80.0-97)
Mean corpuscular hemoglobin concentration	33.4 g/dL (32-36)
Red cell distribution width	15.8% (11.6-14)
Platelets	243.000 mL (140.000-440.000)
Prothrombin time	10.7 seconds (9.0-13.0)
Activated partial thromboplastin time	33.0 seconds (25.1-36.5)
Urea	31 mg/dL (18-55)
Creatinine	0.58 mg/dL (0.7-1.3)
Iron	28 ug/dL (65-75)
Iron-binding capacity	271 ug/dL (69-240)
Ferritin	11.9 ng/mL (21.8-274.6)
Vitamin B12	336.0 pg/mL (187-1059)
Folic acid	7.7 ng/mL (5.3-14.4)

An endoscopy of the upper gastrointestinal tract, extending to the second portion of the duodenum, was performed and showed no abnormal findings. A colonoscopy up to the ileocecal valve also revealed no significant lesions.

**Figure 1:**
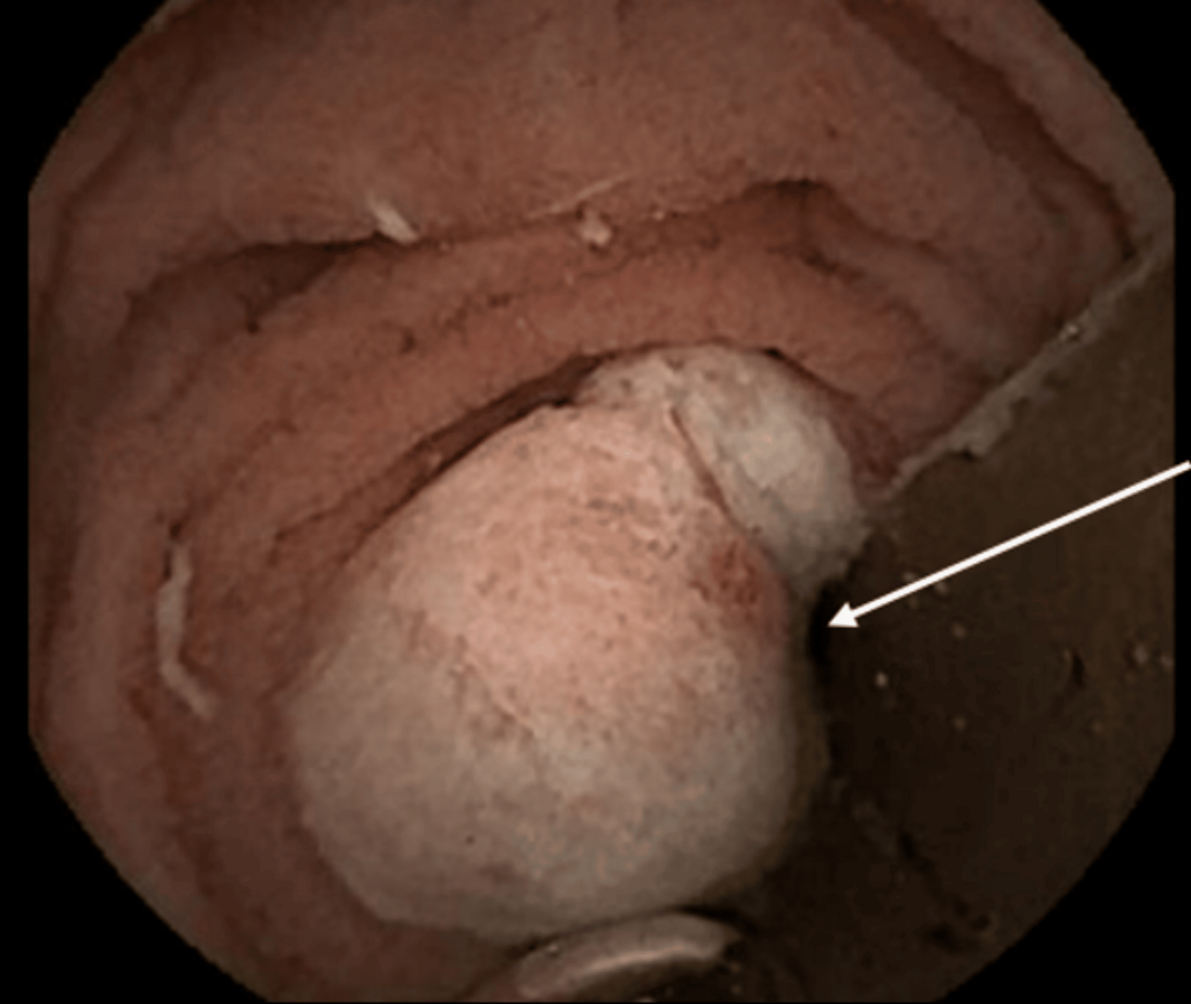
Video capsule endoscopy showing an ulcerated lesion in the proximal jejunum

**Figure 2:**
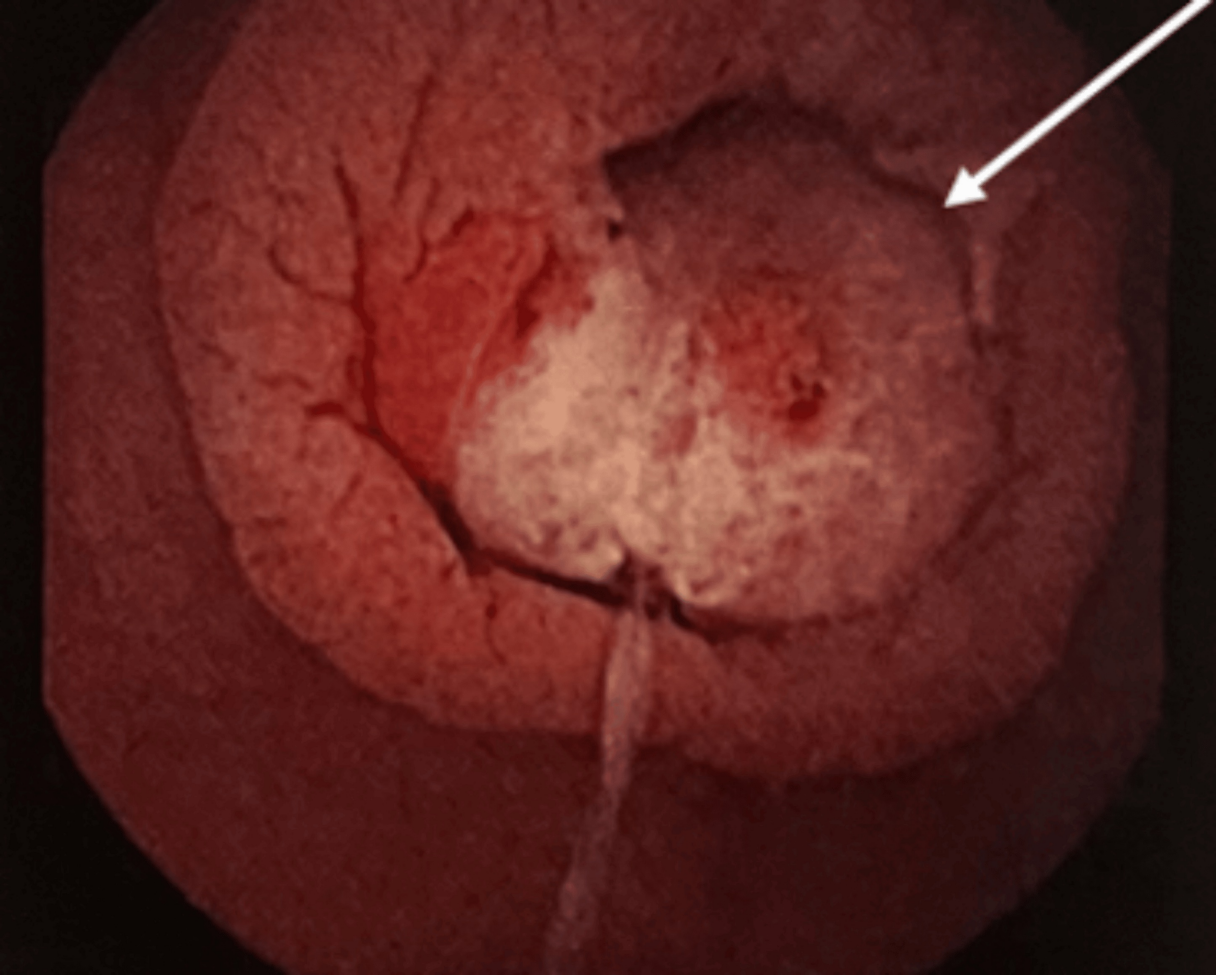
Double balloon enteroscopy showing endoluminal lesion in the proximal jejunum. Biopsies were performed and the lesion was marked with ink

**Figure 3:**
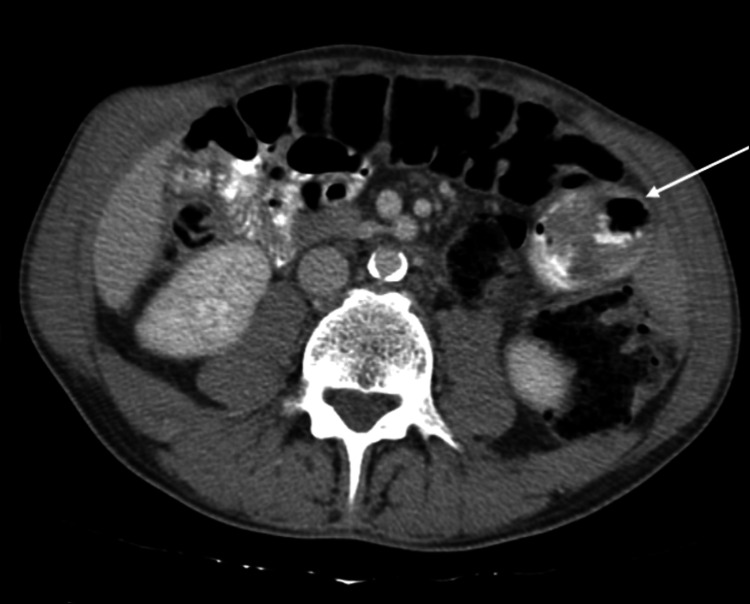
Abdominal CT demonstrating an endoluminal jejunal lesion, which was later confirmed to be jejunal adenocarcinoma (axial view)

**Figure 4:**
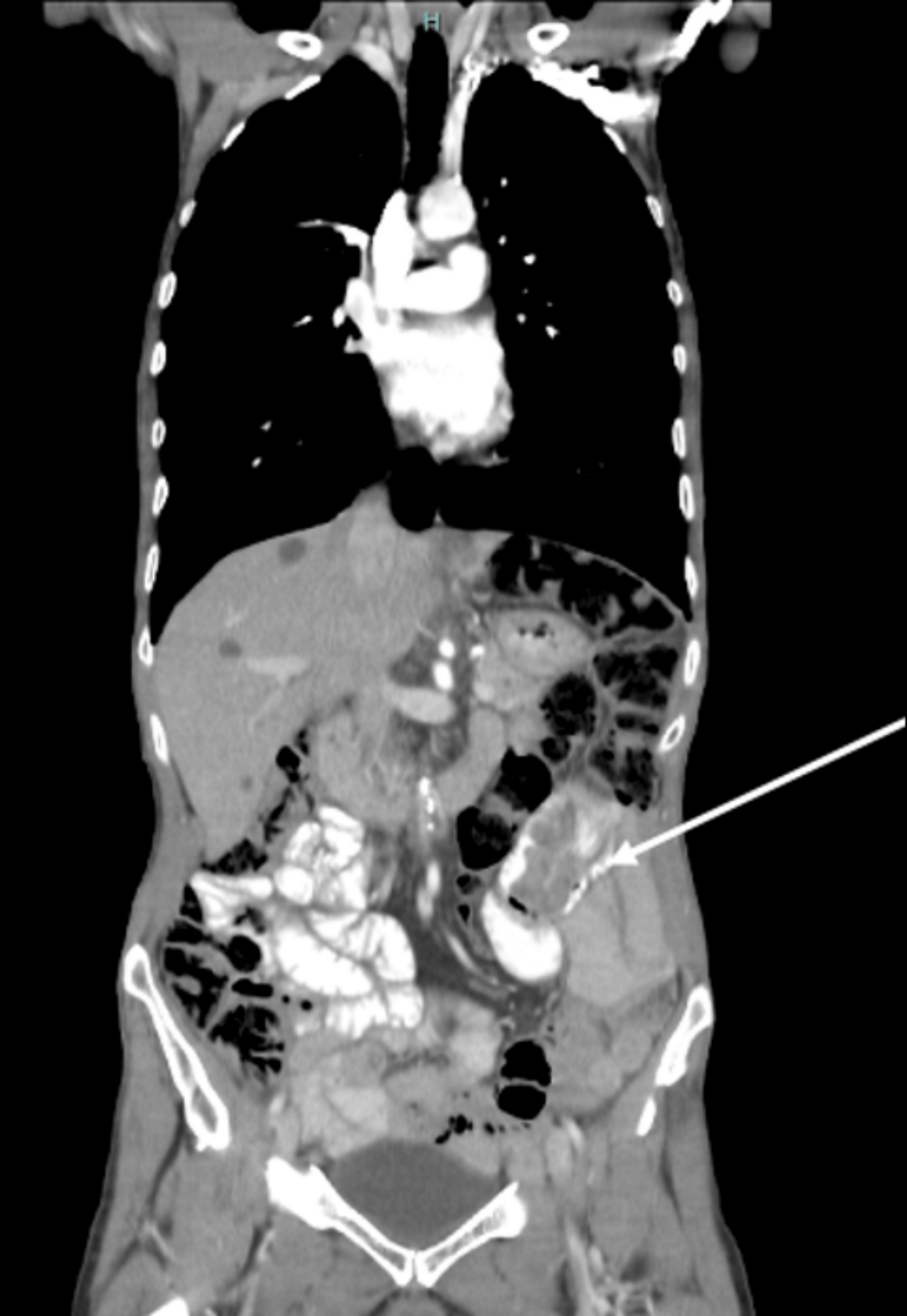
Abdominal CT demonstrating an endoluminal jejunal lesion (coronal view)

Due to the persistent anemia, which could potentially indicate an early sign of gastrointestinal malignancy, a video capsule endoscopy (Figure 1) was conducted, which revealed stenosis due to an ulcerated lesion in the proximal jejunum. A double-balloon enteroscopy (Figure 2) was subsequently performed, confirming the lesion, and biopsies were compatible with adenocarcinoma. The computed tomography (CT) (Figures 3, 4), confirmed the presence of an endoluminal lesion in the small bowel and excluded the presence of metastatic disease. 

**Figure 5:**
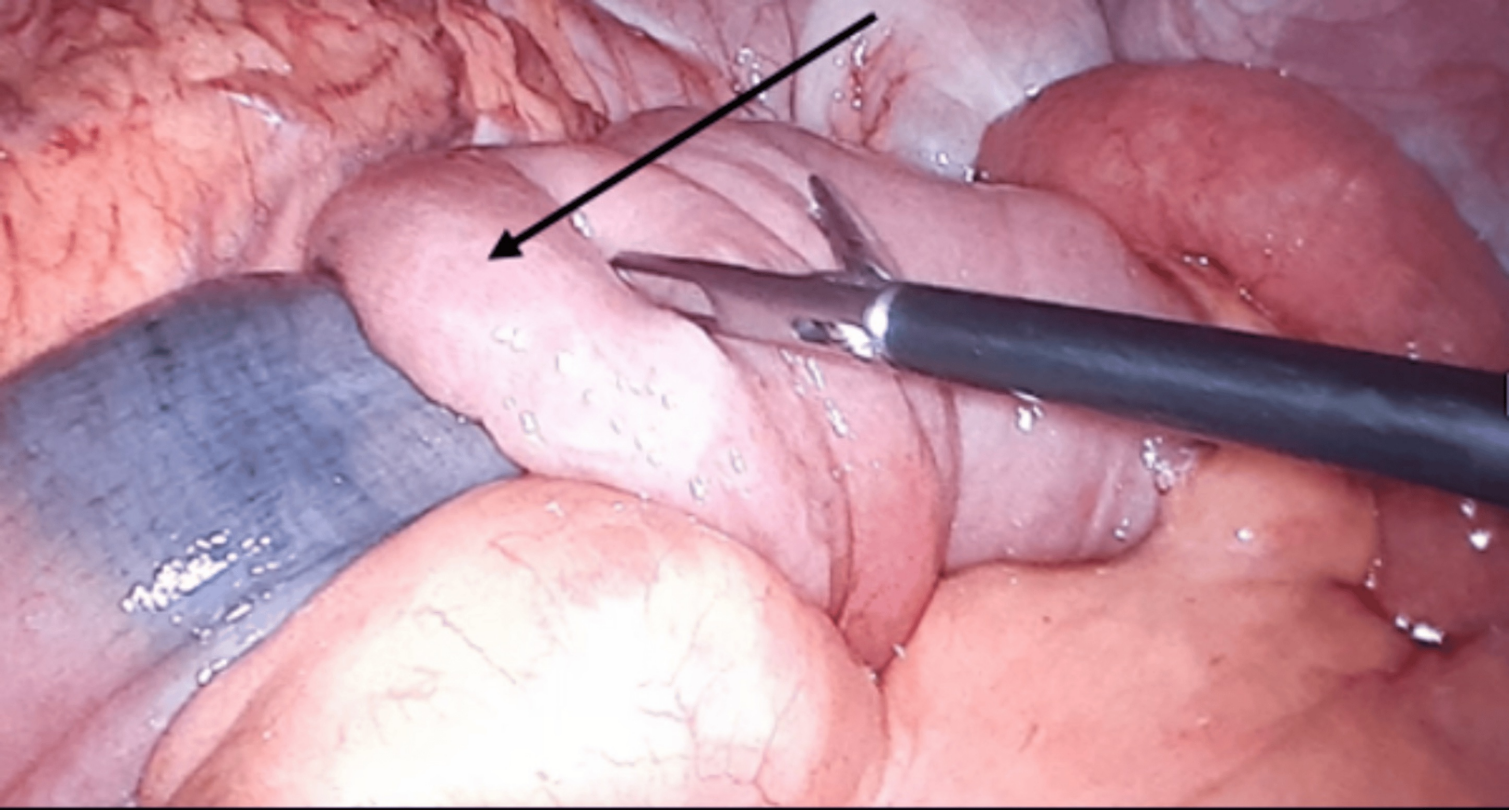
Intraoperative image showing small bowel intussusception

**Figure 6:**
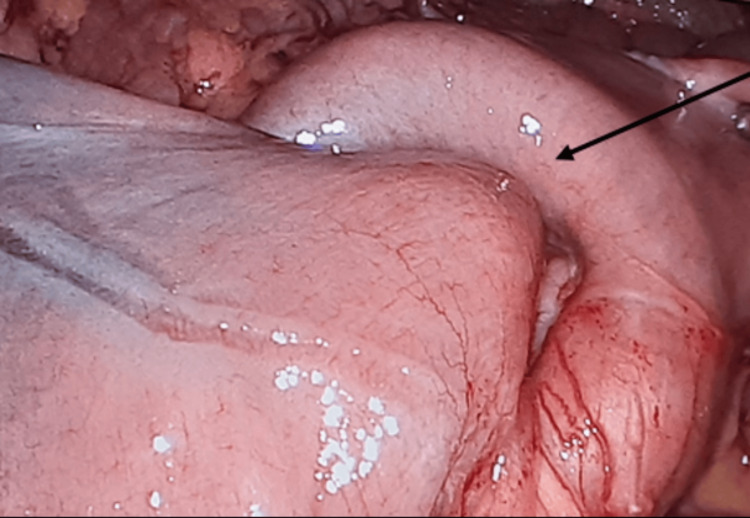
Intraoperative image of the small bowel intussusception, partially reduced

The patient was subjected to an exploratory laparoscopy, which revealed a jejuno-jejunal intussusception (Figures 5, 6) caused by the identified tumor. There were no other relevant findings, such as macroscopic liver and peritoneum metastasis. The intussusception was partially reduced and resection of the affected bowel was carried out, followed by a latero-lateral intracorporeal anastomosis using an EndoGIA^TM^ stapler (Medtronic, Ireland).

The patient had an uncomplicated recovery and was discharged home.

Histopathological examination confirmed a well-differentiated adenocarcinoma with low-grade dysplasia, with no involvement of any lymph nodes (0/6). The tumor staging was pT2N0 according to the TNM classification 9th edition.

## Discussion

The patient initially presented with iron deficiency anemia, resulting from chronic gastrointestinal blood loss due to jejunal adenocarcinoma. During surgery, an intussusception caused by the tumor was discovered, without exhibiting any signs of bowel obstruction.

Adult intussusception is uncommon, accounting for only 1-3% of all cases of bowel obstruction [5]. Unlike in children, where intussusception is typically idiopathic in 90% of cases, an underlying organic cause is found in 90% of adults [3,5]. Malignancy is identified in about 30% of small bowel intussusceptions [6].

Although the precise pathophysiology is not fully understood, one possible explanation is that a lesion within the bowel lumen, combined with peristalsis and the presence of food, causes a narrowing above the lesion and relaxation below it. This dynamic facilitates intussusception [3,5].

In some cases, intussusception presents with nonspecific symptoms such as abdominal pain, nausea, and vomiting, and may manifest as bleeding or abdominal distension. When associated with malignancy, blood-positive stools may appear [7].

Given the nonspecific symptoms and challenges in preoperative diagnosis (58.3%), CT remains the most sensitive diagnostic tool (58-100%) for intussusception [8]. Once identified, the management of adult intussusception usually involves exploratory laparoscopy or laparotomy followed by resection [7]. Resection is crucial to rule out malignancy [8].

In cases involving small bowel malignancies, surgical resection with en-bloc removal of regional lymph nodes provides the best chance for improved survival in patients with localized jejunal adenocarcinoma [2,4]. Although chemotherapy has been explored in some studies, it does not appear to have a significant impact on overall patient survival [4]. As the tumor staging of our patient was pT2N0 (TNM classification 9th edition), there was no indication for chemotherapy.

## Conclusions

Small bowel malignancies are uncommon and often present with few or nonspecific symptoms, making the diagnosis challenging. In some cases, they may present as intussusception.

In this particular case, the patient initially presented with iron deficiency anemia, and during surgery, intestinal intussusception was detected despite the absence of any signs of bowel obstruction. The treatment consisted of surgical resection, and due to the staging of the tumor, there was no indication for adjuvant therapy. After one year of follow-up, there have been no signs of recurrence.

Finally, since iron deficiency anemia may be the only symptom of jejunal adenocarcinoma, clinicians should remain vigilant and initiate further investigation, using advanced imaging techniques like capsule endoscopy when necessary. 
